# A New Approach for Controlling *Agrobacterium tumefaciens* Post Transformation Using Lytic Bacteriophage

**DOI:** 10.3390/plants11223124

**Published:** 2022-11-16

**Authors:** Fiqih Ramadhan, Yuzer Alfiko, Sigit Purwantomo, Andhika Faisal Mubarok, Widyah Budinarta, Antonius Suwanto, Sri Budiarti

**Affiliations:** 1 Graduate School of Biotechnology, IPB University, Bogor 16680, Indonesia; 2 Biotech Laboratory, Wilmar Benih Indonesia, Bekasi 17530, Indonesia; 3 Department of Biology, Faculty of Mathematics and Natural Sciences, IPB University, Bogor 16680, Indonesia

**Keywords:** *Agrobacterium tumefaciens*, lytic bacteriophage, overgrowth

## Abstract

Overgrowth of *Agrobacterium tumefaciens* has frequently been found in *Agrobacterium*-mediated plant transformation. This overgrowth can reduce transformation efficiency and even lead to explant death. Therefore, this research investigates an alternative way to mitigate or eliminate *Agrobacterium* after transformation using a bacteriophage. To develop this alternative method, we conducted effectiveness studies of two lytic bacteriophages (ΦK2 and ΦK4) and performed an application test to control *Agrobacterium* growth after transformation. According to plaque morphological characterization and molecular analysis, the two bacteriophages used in this experiment were distinct. Moreover, some stability physicochemical and growth kinetics, such as adsorption time and susceptibility test, also showed that both bacteriophages differed. On the other hand, the optimum temperature and pH of both phages were the same at 28–30 °C and pH 7. Further investigation showed that both ΦK2 and ΦK4 were able to reduce the overgrowth of *A. tumefaciens* post transformation. Moreover, applying the cocktail (mixture of ΦK2 and ΦK4) with antibiotic application eradicated *A. tumefaciens* (0% overgrowth percentage). This result indicates that the application of bacteriophage could be used as an alternative way to eradicate the overgrowth of *A. tumefaciens* subsequent to transformation.

## 1. Introduction

*Agrobacterium tumefaciens*-mediated transformation is commonly used in plant genetic engineering [1]. This bacterium is widely used because it has several advantages, such as a broad host range of dicots and monocots [2,3,4], with a stable expression of the transferred genes [5,6]. In addition, the process is easy to perform without special supporting tools such as microinjection [1]. Unfortunately, this method has some drawbacks, such as the overgrowth of *Agrobacterium* after callus cocultivation. This overgrowth could lead to a hypersensitivity reaction (HR) and cause tissue browning [7], which could reduce the efficiency of transforming and regenerating plant cells [8].

Several attempts to overcome this problem such as adjusting the pH of the medium, lowering the temperature [9], using an antibiotic in the medium [10,11], and manipulated the *A. tumefaciens* growth by mutation or inserting a certain gene [12,13] have been explored; however, unfortunately, overgrowth still occurs. Therefore, an alternative approach to overcoming overgrowth is required.

Bacteriophages are known as “bacteria eaters” due to their ability to destroy specific host cells [14], requiring certain receptors of the susceptible bacterium to attach to foe infection of the host cell. As natural biocontrols, bacteriophages have several advantages, such as being host-specific, having self-limitation capabilities, mutating following bacterial mutations, being inexpensive to produce, and being found in many environments [15]. Moreover, it is considered not to affect plant growth itself. Therefore, this “assassin machine” of bacteria could be used as an alternative way to control *Agrobacterium* growth.

The potential of bacteriophages as antimicrobial agents has received particular attention due to a wide range of applications such as health, food (agriculture and veterinary), and biotechnology [16]. As an example, bacteriophages have been used to solve problems in the agriculture sector, such as to treat blast disease infection in rice caused by *Xanthomonas oryzae* pv. *oryzae* [17] and wilting disease infection in tomatoes caused by *Ralstonia solanacearum* [18].

Therefore, this research explores the possibility of using bacteriophages to control the *Agrobacterium* growth post transformation. This experiment was divided into three main parts: characterization, stability physicochemical and growth kinetics, and an application test controlling *Agrobacterium tumefaciens* growth post transformation.

This study used two bacteriophage lytic isolates, ΦK2 and ΦK4, with both having specific ranges in *Agrobacterium tumefaciens*. The two bacteriophage isolates were from the bacteriophage collection of PT Wilmar Benih Indonesia, isolated from pond water samples and not previously characterized. For this reason, the two isolates were characterized in this study using morphology, host range, and molecular analysis, followed by effectiveness studies. The effectiveness of phage applications in fighting bacteria depends on several factors, such as the environmental conditions (pH and temperature), phage adsorption rate, burst size, and latent period [19,20,21]. The application test was performed using single bacteriophages or a cocktail of both ΦK2 and ΦK4. The cocktail experiment was performed to mitigate the emergence of bacterial resistance [22].

## 2. Results

### 2.1. Characterization

#### 2.1.1. Morphology Plaque Characterization

**Figure 1:**
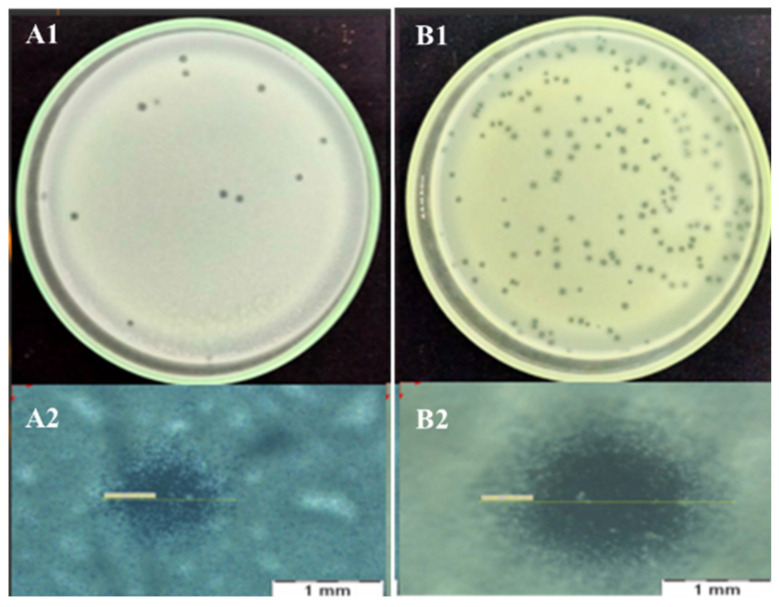
Plaques formed in double-layer agar plates: ΦK2 (**A1**) and ΦK4 (**A2**). Diameter of single plaques: ΦK2 (**B1**) and ΦK4 (**B2**). The bar corresponds to 1 mm.

The plaque indicated that ΦK2 and ΦK4 could be classified as lytic bacteriophages with a transparent appearance (Figure 1). The results of the diameter measurements of ΦK2 and ΦK4 were 1.5 mm and 2.3 mm, with the titer production value of ΦK4 being higher than that of ΦK2 (Table 1). The titer production is essential for controlling the quantity and quality of bacteriophage stock, facilitating determination of the test concentration.

#### 2.1.2. Molecular Characterization Using Restriction Fragment Length Polymorphism (RFLP) and PCR Random Amplified Polymorphic DNA (RAPD)

**Figure 2:**
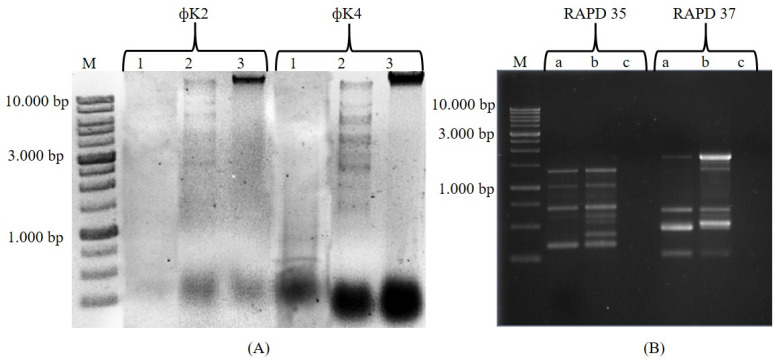
Profile RFLP and PCR RAPD. (**A**) RFLP pattern; M: marker, 1: *Sau*3AI, 2: *Hind*III, 3: un-digested. (**B**) Amplification PCR RAPD; a: ΦK2, b: ΦK4, c: control negative.

Molecular analysis was performed using two different methods, namely, RFLP and RAPD. RFLP assays showed that the restriction enzymes *Eco*RV, *Bam*HI, and *Hae*III were resistant to ΦK2 and ΦK4 genomes, while only *Eco*RI could digest the ΦK4 genome (data not shown). *Sau*3AI and *Hind*III digested both bacteriophage genomes, but the RFLP profile was only formed upon cleavage with *Hind*III (Figure 2A). There were differences in the number of fragments that arose from cutting the genomes of ΦK2 and ΦK4 using *Hind*III, indicating two different bacteriophages. On the basis of the number of bands that appeared and neglecting the possibility of there being two different fragments of the same size, it was estimated that the genome size of ΦK2 was 35 kbp and that of ΦK4 was 56 kbp. The RAPD test showed polymerization between ΦK2 and ΦK4 using both RAPD 35 and RAPD 37 markers (Figure 2B). RAPD 35 showed that ΦK2 successfully amplified four amplicons, while ΦK4 had eight amplicons. For RAPD 37, ΦK2 had four amplicons, and ΦK4 had five.

#### 2.1.3. Host Range Determination

A clear spot due to bacteriophage administration indicated that the bacteria belonged to a certain host range. This test showed that ΦK2 and ΦK4 were classified as bacteriophages with a narrow host range (Table 2). Both bacteriophages could only infect *A. tumefaciens* C58 derivatives, which, in this study, consisted of strains EHA105, AGL1 WT, and AGL1 auxotroph cysteine. Meanwhile, LB4404, a derivative of *A. tumefaciens* Ach5, and an outgroup *E. coli* strain showed no clear spots.

### 2.2. Stability Physicochemical and Growth Kinetics

#### 2.2.1. pH and Thermal Stability

**Figure 3:**
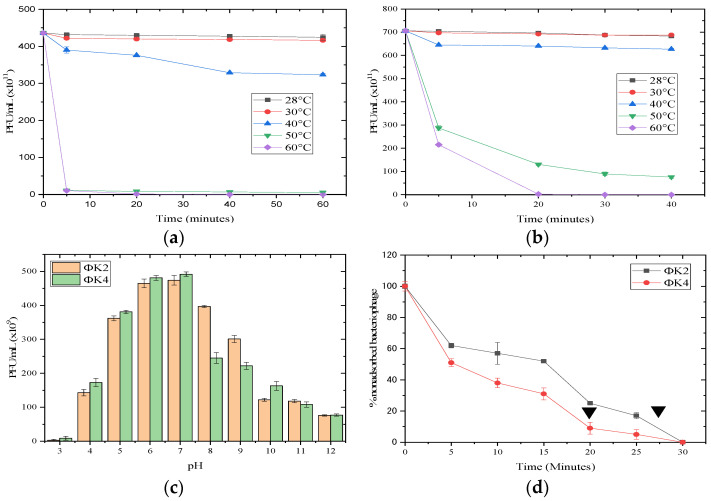
The rate of adsorption and the pH and thermal stability of ΦK2 and ΦK4: (**a**) thermal stability of ΦK2; (**b**) thermal stability of ΦK4; (**c**) pH stability of ΦK2 and ΦK4; (**d**) adsorption of ΦK2 and ΦK4 to the *A. tumefaciens* AGL1 WT host. The black arrow indicates the optimal adsorption value. Results are presented as mean values ± SD.

The pH and temperature stability was measured by calculating the plaque-forming units (PFU) of bacteriophages resistant to the treatment of these physical or chemical parameters. Figure 3a–c show that ΦK2 and ΦK4 had an optimal pH range of 6–7. Furthermore, both bacteriophages had better resistance to alkaline pH conditions than acidic pH. For temperature stability, ΦK2 and ΦK4 had optimal temperatures in the range 28–30 °C. Only in ΦK4 was plaque-forming still detected in the first 20 min of incubation at 50–60 °C.

#### 2.2.2. Adsorption Assay

The two bacteriophages had different optimal adsorption times. The optimal adsorption time was calculated when 90% of bacteriophages were successfully adsorbed; thus, the adsorption times of ΦK2 and ΦK4 were 27 min and 20 min, respectively. Both showed 100% adsorption after 30 min of incubation (Figure 3d).

#### 2.2.3. One-Step Growth

**Figure 4:**
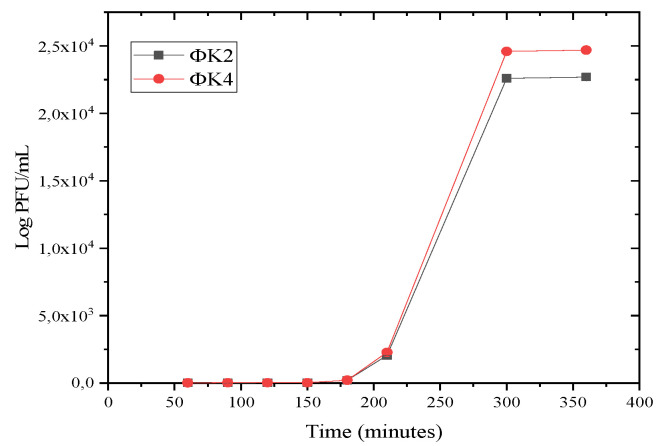
One-step growth curve of ΦK2 and ΦK4. Results are presented as mean values ± SD from three independent experiments.

Figure 4 shows that the latency of the two bacteriophage isolates was observed at 210 min. Meanwhile, the burst size values of ΦK2, and ΦK4, respectively, were 88 PFU per cell and 85 PFU per cell.

#### 2.2.4. Susceptibility Test

In this test, an increase in the value of optical density (OD) over time indicated an increase in the bacterial population. The test results showed that the growth control of *A. tumefaciens* constantly increased with incubation time. Meanwhile, in the application with bacteriophages, *A. tumefaciens* inhibited the growth of *A. tumefaciens* over time.

**Figure 5:**
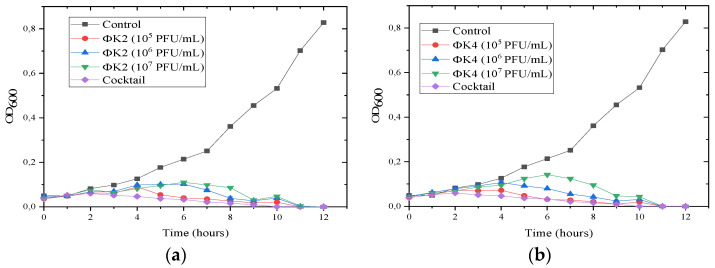
Susceptibility test of ΦK2 and ΦK4 against *A. tumefaciens*: (**a**) ΦK2; (**b**) ΦK4. The treatment cocktail is included in each graphic. Results are presented as mean values ± SD from three independent experiments.

The pattern of inhibition between ΦK2 and K4 differed. For isolate ΦK2, bacterial growth decreased at 7 h of incubation for concentrations of 10^5^ and 10^6^ PFU/mL, while, at 10^7^ PFU/mL, the decrease began to occur at 5 h (Figure 5a). In contrast to ΦK4, at concentrations of 10^5^ PFU/mL, growth inhibition was observed. The *A. tumefaciens* growth decline started at 7 h of incubation, while, at concentrations of 10^6^ and 10^7^ PFU/mL, the *A. tumefaciens* growth decline started at 5 h of incubation (Figure 5b). For cocktails, inhibition began to occur at 3 h of incubation, which was faster than the single treatments (. In general, no treatments showed resistance to *A. tumefaciens*, characterized by no increase in bacterial growth during the 12 h of observation.

### 2.3. Controlling Overgrowth of A. tumefaciens Using Lytic Bacteriophage

#### 2.3.1. Simulation Washing

The simulation stage was critical before testing the application of post-transformation lytic bacteriophages. The length of washing time and the required bacteriophage concentration were determined. The callus washing time determines the success of bacteriophage adsorption on bacteria, in addition to avoiding damage to the callus. The bacteriophage concentration should also be evaluated to consider the possibility of biofilm formation during callus transformation based on *A. tumefaciens*.

This test revealed that both single and cocktail applications were able to completely inhibit the growth of *A. tumefaciens* (Table 3). This inhibition occurred at sampling times of 40 min and 180 min. Therefore, the callus washing time used was 40 min. The aim was to reduce the physical stress on the callus due to prolonged agitation and avoid cross-contamination from other bacteria if the washing process is too long. The bacteriophage concentration used during washing was 10^11^ PFU/mL. This concentration was indeed higher than the simulation concentration so as to avoid adsorption failure and the production of new bacteriophage particles due to the formation of a bacterial biofilm on the callus.

#### 2.3.2. Plant Transformation and Percentage Overgrowth

**Figure 6:**
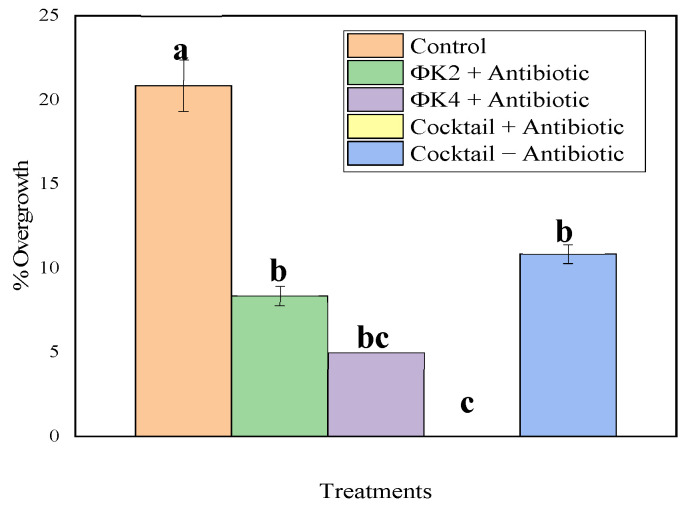
Percentage overgrowth of *A. tumefaciens* on callus. Results are presented as mean values ± SD. The same letters indicate no significant difference between treatments according to the Tukey HSD test at the level of α = 0.05.

The results of the application of overgrowth biocontrol based on lytic bacteriophages can be seen in Figure 6. According to a further Tukey’s test, the overall percentage of overgrowth inhibition treatment (both single and cocktail treatments) was significantly different compared to the control (Tukey’s test, *p* < 0.05). At this stage, we also modified one of the treatments by using only the phage cocktail and eliminating the washing process using antibiotics. This test also yielded results that were significantly different from the control. The observation results show that the percentage of overgrowth inhibition was as follows: control (20.83%); ΦK2 + antibiotic (8.33%); ΦK4 + antibiotics (5.00%); cocktail + antibiotic (0.00%); cocktail − antibiotic (10.83%).

## 3. Discussion

Water is a great source of bacteriophages, with several studies reporting the isolation of phages from water samples such as wastewater [23,24], seawater [25,26], freshwater [27,28,29,30,31], pond water [32,33], and even extreme aquatic environments such as geothermal waters [34]. Similarly, the bacteriophage isolate used in this study was from the PT Wilmar Benih Indonesia culture collection, and it was isolated from the pond water samples in the Cikarang (−6.286442338899404, 107.13851016995596) and Tangerang Selatan (−6.334631104794968, 106.75284144333432). The two isolates were coded as ΦK2 and ΦK4.

As these bacteriophages were not previously studied, we characterized them in this study. The results of the plaque-forming unit characterization showed that both bacteriophages had a clear bacteriophage appearance and no halo; hence, they could be classified as lytic bacteriophages. The bacteriophage criterion for biocontrol is the lytic type, where the bacteriophage can directly enter the lytic phase without entering the lysogenic stage. In the lysogenic phase, the bacteriophage integrates its genetic material with the host cells. One of the consequences is the development of resistance properties in bacteria [35,36]. In the tailed bacteriophage type, the lysis mechanism is controlled by two main components, namely, lysin (an enzyme capable of cleaving one essential bond in the peptidoglycan matrix) and holin (a protein attached to a pore in the inner membrane that serves to provide the right time for lysin to reach the peptidoglycan layer and accelerate lysis) [37]. The plaque sizes of ΦK2 and ΦK4 were different. According to the classification of Ellis and Winters [38], ΦK2 plaques could be classified as small plaques (less than 2 mm), while ΦK4 was classified as a bacteriophage with large plaques (more than 2 mm). This difference could be used to distinguish between the two phages. The plaque-forming size is generally small in the Myoviridae family compared to the Siphoviridae and Podoviridae families. The small plaques of the Myoviridae family have a large capsid, resulting in a slower diffusion rate into bacterial cells compared to the Siphoviridae and Podoviridae families [39].

RFLP analysis showed that distinct polymorphisms were only detected in ΦK2 and ΦK4 using *Hind*III. Other enzymes such as *Eco*RV, *Bam*HI, and *Hae*III could not digest the ΦK2 and ΦK4 genomes (data not shown). The condition of bacteriophages being refractory to restriction enzymes has been widely reported. For example, DNA samples of Siphoviridae phages infecting *Leuconostoc fallax* were highly refractory to digestion by several endonucleases, including *Alu*I, *Bam*HI, *Eco*RI, *Hind*III, *Rsa*I, and *Sau*3AI [40]; genomes of ΦSt2 and ΦGrn1 infecting *Vibrio alginolyticus* could not be digested by *Hpa*II, *Hae*III, and *Bam*HI [41] This condition can occur for several reasons, including unusual base integration in viral DNA [42], such as hydroxymethyl uracil or hydroxymethyl cytosine, as well as the natural loss of restriction sites during evolution [43]. Furthermore, it is hypothesized that there is an adaptive mechanism involving the loss of restriction sites [42] and the transfer of methylase genes in the phage genome [44]. From the results of the *Hind*III digestion profile, it was estimated that the genome for ΦK2 was 35 kbp and that for ΦK4 was 56 kbp. Both bacteriophages were classified as *dsDNA* (double-stranded DNA). Bacteriophages with *dsDNA* are more commonly found than other groups [45]. Phages with *dsDNA* belong to the order Caudavirales, which includes three phage families, Myoviridae, Siphoviridae, and Podoviridae [46].

RAPD analysis of phage DNA provided a simple and reproducible method for DNA fingerprinting. This method has been used extensively for microbial characterization, differentiation of closely related bacteriophages species, and identification of strain poly-morphisms [47]. RAPD analysis of ΦK2 and ΦK4 using markers RAPD35 and RAPD37 displayed a polymorphic amplification. This result supported the previous characterizations indicating differences in bacteriophages ΦK2 and ΦK4. The use of RAPD to determine the diversity of bacteriophages was previously carried out in studies such as populations of lytic bacteriophages *P. aeruginosa* and *S. aureus* [48] and the various phages that infect *E. coli* [49].

*A. tumefaciens* can generally be divided into two groups according to its chromosomal and Ti plasmid presence, i.e., strains C58 [50] and Ach5 [51]. The *A. tumefaciens* strains used in this study, namely, AGL1 WT, AGL1 auxotroph, AGL1-eGFP, and EH105, were classified as C58 non-opaline, and the Ti plasmid was modified from the plasmid *pTiBo542* or *pTiC58* [52,53,54,55]. Meanwhile, strain LB4404, which cannot be infected, are classified as opaline type Ach 5 and have Ti plasmid *pAL4404* [56]. For the outgroup host range, ΦK2 and ΦK4 were unable to infect strains of *E. coli* bacteria. The same phenomenon was also reported by another study, where ΦAtu_ph04 dan ΦAtu_ph08 caused lysis of most C58-derived *A. tumefaciens* strains [57], while ΦAtu_ph02 and ΦAtu_ph03 only had a host range incorporating the C58-derived *A. tumefaciens* group [58].

Temperature and pH factors are physiochemical factors that can affect the effectiveness of bacteriophage application against pathogenic bacteria [21]. Generally, high temperature conditions can result in permanent damage or denaturation of virus particles [59]. The stability of these physical parameters determines the success of bacteriophage adsorption during callus application. The condition of the plant callus washing process is often at pH 7 and in a temperature range of 25–28 °C [60].

The adsorption time is a factor that determines the success of bacteriophage infection [61]. Here, ΦK2 and ΦK4 had slightly different adsorption times. At 5 min prior to infection, a sharp decline in bacteriophage adsorption occurred, where ΦK4 showed higher bacteriophage adsorption with an 11% difference compared to ΦK2. The different adsorption rates in the first five minutes affected the optimal adsorption time, with ΦK2 at 27 min and ΦK4 at 20 min. This result also supported the characterization results highlighting differences in the bacteriophages.

The susceptibility test of the bacteriophages showed great potential for ΦK2 and ΦK4 in inhibiting the growth of *A. tumefaciens* AGLI. Both bacteriophages used in this experiment showed great lytic activity, with a slightly different inhibition time. The main difference was at the concentration of 10^6^ PFU/mL, where the lytic activity of ΦK4 started earlier than that of ΦK2 at 5 h and 7 h, respectively. This indicates that ΦK4 had greater inhibitory properties than ΦK2. Moreover, our results showed a similar pattern to other bacteriophages, where a higher concentration value led to earlier bacterial growth inhibition [62]. Interestingly, in both ΦK2 and ΦK4, there was no sign of bacteriophage-resistant bacteria emerging during the 12 h of observation. Nevertheless, to eliminate the possibility of phage-resistant bacteria, a phage cocktail was also tested, showing tremendous bacterial growth inhibition compared to the single applications, with bacterial growth starting to decline at 3 h post inoculation. There are several advantages of cocktail applications, such as broader-spectrum activity [63,64] and suppression of the development of phage-resistant bacteria [65,66]. Cocktail-based bacteriophage therapy is widely used to treat bacterial infections of plant pathogenic bacteria such as *P. carotovorum* subs. *carotovorum*, *P. syringae* pv. *porri*, and *D. solani* [67,68,69].

Different simulation results were possible within one application because the bacteria were in free or planktonic conditions. However, in the washing step, *A. tumefaciens* formed a biofilm that infected the callus. In *A. tumefaciens*, the change from a planktonic free-floating phase to biofilm formation in the attachment process and the provision of structural stability are regulated by the production of exopolysaccharide (EPS). In addition, virulence factors such as swimming motility, exoprotease production, and cell surface hydrophobicity affect biofilm formation in *A. tumefaciens* [70,71,72]. The formation of this biofilm makes it difficult for bacteriophages to infect bacteria. This biofilm structure is resistant to various types of stress, including bacteriophage predation. This difference is due to the condition where the bacterial biofilm is formed, making it difficult to penetrate the biofilm layer, thus inhibiting phage productivity during infection [73,74]. In addition, the bacteria were stationary in the biofilm condition; thus, it was more challenging to infect phages than when they were in an exponential condition [75]. In planktonic conditions, the bacteria are free-floating; therefore, the number of bacteria with the potential to contact the environment is greater than the biofilm [76]. The efficacy of bacteriophages against planktonic cultures is much greater than under biofilm formation conditions. Several studies have also reported that phages are more effective in fighting against planktonic *staphylococcal* infections [77], and that bacteriophages are effective on planktonic *S. epidermidis* planktonic cells [78]. One alternative to overcome the formation of biofilms is the use of cocktails. Cocktail bacteriophages, in their application, are more effective than single phages due to their more comprehensive range of activity and a lower likelihood of developing phage resistance through bacterial biofilms [79]. The latter is caused by callus washing application with phages; the percentage of *A. tumefaciens* overgrowth could be suppressed maximally in the cocktail treatment compared to a single application.

A positive result in the overgrowth suppression test is also possible as a function of the interaction mechanism between phage and antibiotics. This mechanism is known as phage–antibiotic synergism (PAS). The presence of PAS inhibits the growth of bacteria due to the bactericidal properties of antibiotics with lytic bacteriophages [80]. The PAS interaction in this study occurred between ΦK2/ΦK4 and cefotaxime. Although hygromycin represents a group of antibiotics, in the case of plant transformation, hygromycin exists as a selective marker, not as a bactericidal agent. In this study, the hygromycin resistance gene/hpt gene (hygromycin phosphotransferase) existed on the plasmid *pCAMBIA1300-eGFP-hpt*. The effectiveness of cefotaxime and phages in reducing bacterial growth was also reported in other studies, including the combination of ΦMRM57 with cefotaxime in suppressing *Citrobacter amalonaticus* infection [81], and the combination of isolate ΦCpl-711 with amoxicillin or cefotaxime in suppressing the serotype 23F multidrug-resistant clinical isolate of *S. pneumoniae* [82]. The results of a comparison with previous studies using the *A. tumefaciens* AGL1 auxotroph mutant [12] allows concluding that the suppression value of overgrowth is as follows: control > auxotroph > cocktail without antibiotic > individual bacteriophage + antibiotic > cocktail + antibiotic.

## 4. Materials and Methods

### 4.1. Characterization

#### 4.1.1. Morphology Plaque and Quantification Bacteriophage

Characterization of plaque morphology refers to Topka et al. [83] with minor modifications. Bacteriophage stock was diluted using CaCl_2_/MgCl_2_ (1 M CaCl_2_, 80 mM MgCl_2_) buffer, and then 100 µL of the dilution was mixed with 100 µL of *A. tumefaciens* bacteria before adding 4.8 mL of top agar (0.2% (*w*/*v*)) yeast extract; 5% (*v*/*v*) glycerol; 1.1% (*w*/*v*) CaCl2, 0.4%(*w*/*v*) agar). Top agar was applied in a standard double layer above basal LA (1% (*w*/*v*) yeast extract; 4% (*w*/*v*) tryptone; 4% (*w*/*v*) NaCl, 5% (*v*/*v*) glycerol). Calculation of plaque size was performed using an Olympus SZX16 microscope.

Quantity calculations were carried out following the double-layer standard described above. After incubation, only plates with plaque formation ranging from 30 to 300 were accepted. The bacteriophage concentration was calculated using the formula phage concentration (PFU/mL) = a number of plaques × 10 × reciprocals of counted dilution [84].

#### 4.1.2. Restriction Fragment Length Polymorphism (RFLP) and PCR Random Amplified Polymorphic DNA (RAPD)

The bacteriophage genome was isolated using the Wizard^®^ Genomic DNA Purification Kit (Promega) according to the manufacturer’s protocol with slight modification. RFLP uses several restriction enzymes, including *Eco*RI, *Eco*RV, *Bam*HI, *Hae*III, *Hind*III, and *Sau*3AI (New England Biolabs [NEB]). Restrictions followed the manufacturer’s protocol; DNA (1 µg/µL) was digested individually with 10 U of enzyme in a total reaction of 20 µL and incubated at 37 °C for 3 h. After incubation, the restriction results were electrophoresed using 1% (*w*/*v*) agarose at a voltage of 80 V for 60 min and stained with ethidium bromide. The agarose gel was then visualized with UV Transilluminator gel (Gel Doc XR+ System, Bio-Rad Laboratories Inc., Heracles, CA, USA).

RAPD markers were performed by amplifying DNA using GoTaq Green Master Mix (Promega) in a 10 µL reaction with the following composition: 1 µL of DNA template (5 ng/μL), 5 µL of master mix, 1 µL of primer, and 3 µL of nuclease-free water (Promega). The PCR conditions for RAPD35 (CCGCAGCCAA) and RAPD37 (AACGCGCAAC) were as follows: pre-denaturation at 95 °C, 3 min; denaturation at 95 °C, 3 s; annealing at 48 °C, 30 s; extension at 72 °C, 1 min; post extension at 72 °C, 8 min. The PCR results were visualized using electrophoresis following the same method as above.

#### 4.1.3. Host Range Determination

Host range testing using the spot test method refers to Narulita et al. [85]. The bacteria used in the test included two genera: *Agrobacterium* and *Escherichia* (Table 2). Specifically, 100 µL of bacteria were inoculated in 4.8 mL of top agar. Then, 5 µL of bacteriophage was dripped onto the solidified top agar medium. The plates were incubated at the optimum temperature and incubation time for each bacterium, *A. tumefaciens* (28 °C, 72 h) and *E. coli* (37 °C, 24 h). Furthermore, observations were made and classified as a function of the degree of spot brightness into two different classes: (+) bacteriophage-susceptible; (−) bacteriophage-resistant [84].

### 4.2. Stability Physicochemical and Growth Kinetics

#### 4.2.1. pH and Thermal Stability

A total of 100 µL of bacteriophage was added to 900 µL of LB, adjusting the pH from 3 to 12. The mixture was incubated at 28 °C for 60 min and continued with a double layer. To test the temperature stability of bacteriophage stock in CaCl_2_/MgCl_2_ buffer, it was incubated at 28 °C, 30 °C, 40 °C, 50 °C, and 60 °C for 60 min. Furthermore, plaque calculations were performed using a standard double layer [86].

#### 4.2.2. Adsorption Test

The adsorption kinetics test used *A. tumefaciens* AGLI (10^8^ CFU/mL) and bacteriophage (10^3^ PFU/mL). The mixture was incubated at 28 °C. Subsequently, sampling was performed at 0, 5, 10, 15, 20, 25, and 30 min and centrifuged at 13,000× *g* for 10 min to separate the adsorbed bacteriophages. Then, 100 µL of supernatant was taken, double-layered, and counted for non-adsorbed bacteriophage particles. The number of non-adsorbed bacteriophages and bacterial hosts at time 0 was 100% [87].

#### 4.2.3. One-Step Growth

Bacteria that entered the mid-log phase of 10^8^ CFU/mL were mixed with bacteriophages (10^3^ PFU/mL), and then left for 10 min for adsorption onto the bacterial cell. Then, 1 mL of the mixture was taken and put into 9 mL of medium in a new flask and left for another 10 min. The same procedure was repeated until the dilution was 10^−4^. The mixed cultures were incubated at 28 °C for 6 h. Samples of up to 100 µL were taken from each flask at a predetermined time to avoid the PFU/mL value being too numerous to count [88]. The burst size value was calculated by dividing the average PFU/mL plateau period by the average PFU/mL latent period [89].

#### 4.2.4. Susceptible Test

Several concentrations of bacteriophage were used to calculate the susceptibility of each isolate according Muturi et al. [90] with minor modifications. Bacteriophages with concentrations of 10^5^, 10^6^, or 10^7^ PFU/mL and *A. tumefaciens* (10^6^ CFU/mL) were mixed, whereas the cocktail treatment was performed using a bacteriophage concentration of 10^7^ PFU/mL. As a control, LB was only inoculated with bacteria. All treatments were incubated at 28 °C and observed for 12 h. The OD_600_ was observed every 1 h interval using a 96-well plate. All treatments were repeated three times.

### 4.3. Controlling Overgrowth of A. tumefaciens Using Lytic Bacteriophage

#### 4.3.1. Simulation Washing

This method was developed by PT Wilmar Benih Indonesia, Cikarang-Indonesia. *A. tumefaciens* bacteria were cultured overnight and then diluted with sterile distilled water to obtain an OD_600_ = 0.01 (10^6^ CFU/mL). Next, a mixed culture was generated with bacteriophages at several concentration values, namely 10^7^, 10^8^, and 10^9^ PFU/mL. The mixed culture was then incubated for 180 min. The mixed culture measurements were taken at 40 and 180 min. A total of 1 mL of mixed culture was taken at each predetermined sampling time, and then a plaque assay was performed by pouring 4 mL of top agar over the basal LB. Overgrowth was indicated by counting the number of colonies that grew on the medium after incubation for 72 h at 28 °C. Tests were also carried out using a cocktail of ΦK2 and ΦK4 at 10^8^ PFU/mL with a 1:1 (*v*/*v*) ratio. The test was carried out using three individual replications.

#### 4.3.2. Plant Transformation and Percentage Overgrowth

Transformation explants used calluses derived from the scutellum of the Nipponbare rice plant [60]. A total of 40 calluses for each replication were infected for 1.5 min using a suspension of *A. tumefaciens* OD_600_ = 0.1 carrying the plasmid *pCAMBIA1300-eGFP-hpt* in 50 mL of AAM + 100 mM acetosyringone medium (USA) [91]. For the cocultivation process, the callus was placed on the surface of sterile filter paper and then transferred to N6D-AS medium. The callus was incubated in the dark at 25 °C for 3 days.

According to Tamzil et al. [12], the callus washing process was carried out with slight modifications. The first wash used sterile water five times for 5 min each. The second wash was carried out using sterile water, and cefotaxime (300 mg/L) was added for 15 min. Subsequently, the callus was washed with a single application or cocktail 1:1 (*v*/*v*) with a phage cell density of 10^11^ PFU/mL for 40 min. For the control treatment, washing was carried out using only phage buffer, i.e., CaCl_2_/MgCl_2_ (1 M CaCl_2_, 80 mM MgCl_2_) buffer; the treatment details are shown in Table 4. The callus was then dried for 30 min on sterile filter paper and transferred to N6D-CH medium (cefotaxime 300 mg/L and hygromycin 50 mg/L). The callus was incubated under constant lighting conditions at 25 °C for 4 weeks. Specifically, the cocktail treatment without cefotaxime antibiotic was not added to the second washing process or the selection medium, and 5 µL of cocktail suspension was added for each callus.

The number of contaminated calluses was observed for 4 weeks. Furthermore, the percentage overgrowth was calculated using the following formula: percentage contamination = (number of calluses contaminated)/(total number of calluses) × 100%. The data were analyzed using one-way ANOVA followed by a Tukey HSD test (α = 0.05). All data were visualized using Origin Pro 2019b software (Origin Lab Corp., Northampton, MA, USA).

## 5. Conclusions

In this paper, we concluded that the two bacteriophage isolates were different on the basis of several characterization results. As a function of the morphological plaque, both ΦK2 and ΦK4 were categorized as lytic bacteriophages with different plaque sizes (ΦK2, 1.5 mm; ΦK4, 2.3 mm). Furthermore, the molecular analysis supported the morphological observation result, with both phages showing a different pattern of RFLP and RAPD.

The effectiveness of phage applications depends on several factors, such as the environmental conditions (pH and temperature), phage adsorption rate, burst size, and latent period. The optimal temperature and pH of isolates were at 28–30 °C and pH 7. The optimal adsorption times for ΦK2 and ΦK4 were 27 min and 20 min, respectively. In addition, a susceptibility test showed that ΦK4 had greater inhibitory properties than ΦK2. The information gained in the effectiveness studies was used for the application test.

In the application test, the percentage overgrowth showed that ΦK2 and ΦK4 or a cocktail could reduce or even eliminate the *A. tumefaciens* growth post transformation. During the single application test, ΦK2 and ΦK4 could significantly reduce the overgrowth compared to the control, and there was no significant difference between ΦK2 and ΦK4. Interestingly, the application of a cocktail without antibiotics significantly reduced the *Agrobacterium* growth compared to the control. This phage cocktail application could banish the side-effects of antibiotic application. Nevertheless, the highest suppression was shown in the cocktail treatment combined with antibiotics (percentage overgrowth of 0%). This result indicates that bacteriophage application could be used as an alternative to eradicate the *Agrobacterium tumefaciens* overgrowth post transformation.

## Figures and Tables

**Table 1:** Plaque morphological characteristics of bacteriophages.

Phage	Plaque Morphology	Plaque Diameter (mm)	Titer Phage (PFU/mL)
ΦK2	Clear, without halo	±1.5	5.4 × 10^17^
ΦK4	Clear, without halo	±2.3	7.8 × 10^17^

**Table 2:** Host spectrum of ΦK2 and ΦK4.

Bacterial Strain	Isolate Bacteriophage	
	ΦK2	ΦK4
*Agrobacterium tumefaciens*		
AGL1 WT	+	+
AGL1 Auxotroph Cystein	+	+
EHA105	+	+
LB4404	−	−
*Escherichia coli*		
DH5α	−	−
Top10	−	−
BL21 (DE3)	−	−

(+) bacteriophage-susceptible; (−) bacteriophage-resistant.

**Table 3:** Determination of minimal concentration and washing time of bacteriophage.

Bacteriophage Concentration (PFU/mL)	ΦK2		ΦK4		Cocktail	
	40 min (Mean ± SD)	180 min (Mean ± SD)	40 min (Mean ± SD)	180 min (Mean ± SD)	40 min (Mean ± SD)	180 min (Mean ± SD)
10^7^	0 ± 0	0 ± 0	0 ± 0	0 ± 0	0 ± 0	0 ± 0
10^8^	0 ± 0	0 ± 0	0 ± 0	0 ± 0	0 ± 0	0 ± 0
10^9^	0 ± 0	0 ± 0	0 ± 0	0 ± 0	0 ± 0	0 ± 0

**Table 4:** Treatment details for overgrowth observation on rice callus.

Detail Treatment	Control	ΦK2 + Antibiotic	ΦK4 + Antibiotic	Cocktail + Antibiotic	Cocktail − Antibiotic
Washing step					
Cefotaxime 300 mg/L	√	√	√	√	×
CaCl_2_/MgCl_2_	√	×	×	×	×
Titer phage (10^11^ PFU/mL)	×	√	√	√	√
Selection medium					
Cefotaxime 300 mg/L	√	√	√	√	×
5 μL drop of phage cocktail	×	×	×	×	√

## Data Availability

The data of this study are available within the article.
